# Crack Detection of Threaded Steel Rods Based on Ultrasonic Guided Waves

**DOI:** 10.3390/s22186885

**Published:** 2022-09-12

**Authors:** Kunhong Peng, Yi Zhang, Xian Xu, Jinsong Han, Yaozhi Luo

**Affiliations:** 1 College of Civil Engineering and Architecture, Zhejiang University, Hangzhou 310058, China; 2 Center for Balance Architecture, Zhejiang University, Hangzhou 310058, China; 3 College of Computer Science and Technology, Zhejiang University, Hangzhou 310027, China

**Keywords:** ultrasonic guided wave, threaded rods, longitude mode, crack detection

## Abstract

Fatigue cracks are typical damage of threaded steel rods under dynamic loads. This paper presents a study on ultrasonic guided waves-based, fatigue-crack detection of threaded rods. A threaded rod with given sizes is theoretically simplified as a cylindrical rod. The propagation characteristics of ultrasonic guided waves in the cylindrical rod are investigated by semi-analytical finite element method and the longitudinal L(0, 1) modal ultrasonic guided waves in low frequency band is proposed for damage detection of the rod. Numerical simulation on the propagation of the proposed ultrasonic guided waves in the threaded rod without damage shows that the thread causes echoes of the ultrasonic guided waves. A numerical study on the propagation of the proposed ultrasonic guided waves in the threaded rod with a crack on the intersection of the smooth segment and the threaded segment shows that both linear indexes ($R_{f}$ and *ARS*) and nonlinear indexes ($\beta_{re}^{\prime }$ and $\beta^{\prime }$) are able to detect the crack. A constant-amplitude tensile fatigue experiment was conducted on a specimen of the threaded rod to generate fatigue cracks in the specimen. After every 20,000 loading cycles, the specimen was tested by the proposed ultrasonic guided waves and evaluated by the linear indexes and nonlinear indexes. Experimental results show that both the linear and nonlinear indexes of the ultrasonic guided waves are able to identify the crack before it enters the rapid growth stage and the nonlinear indexes detect the crack easier than the linear indexes.

## 1. Introduction

Threaded steel rods are widely used in engineering structures as load-bearing members or connections such as bridge suspenders, tendons and bolts. These members are usually under high stress and vulnerable to defects and fatigue cracks under periodic dynamic loads [1]. Failure of them will result in safety accidents and economic losses. Nondestructive damage detection of in-service threaded steel rods is a useful way to avoid sudden failure of them.

There are various nondestructive damage detection approaches, such as magnetic particle testing [2,3], eddy current sensing [4,5], acoustic emission [6,7], vibration-based inspection [8,9,10], and ultrasonic testing [11,12,13,14] for steel members. The magnetic-particle method relies on the interaction between magnetic powder and defects. Although its efficiency is improved with modern intelligent magnetic-particle machines [2], the detection process is still cumbersome due to unavoidable procedures such as surface cleaning, magnetization and demagnetization [3]. In addition, it has different sensitivity to the defection in a different magnetization direction, which makes it is difficult to find circumferential cracks paralleling the threads [14]. The eddy current method, which is based on the change of voltage or current in sensing coil, is efficient and sensitive to circumferential cracks. However, the defect at the root of the threads usually causes special signals that are difficult to process [4] and the signal lags severely as the detection depth increases [5]. The acoustic emission method, which judges and locates damage by capturing the high-frequency elastic waves generated by the release of energy when damage occurs, has high spatial resolution and sensitivity [6]. It can be used to detect metal cracks even if the acoustic emission is relatively weak [7]. However, there are some weaknesses in this method that need our attention such as complex cabling and high sensitivity to environmental noise [6]. The vibration-based inspection method, which detects damage through changes of dynamic properties, has been used to detect and localize multiple cracks in uniform rods [8] and non-uniform rods [9]. However, it is vulnerable to the noise, and external factors such as inconsistent boundary conditions and environment changes will affect the stability and accuracy of the detection [10].The ultrasonic method is well-developed and has high-efficiency [13]. It mainly uses straight probes and small-angle oblique, identifying damage from the waveform changes [11,12]. However, the ultrasonic signal will be interfered with by boundary and variable cross-section contour boundary, resulting in disturbance wave, deformation wave, etc., which lowers the effectiveness of damage detection [14]. Moreover, Single probe angle, detection space limitation and poor sonic accessibility also bother the users [14]. Ultrasonic guided waves (UGWs) are a kind of ultrasonic waves that propagate over the entire cross section of the components. Compared with the traditional high-frequency ultrasonic testing, it has lower frequency and is more sensitive to frequency. Its low attenuation and high propagation distance make it possible for long-range and hidden-area detection [15,16]. 

The UGW method has already been applied for damage inspection of pipes [17], composite plates [18], cables [19], and other types of structural components [20]. In particular, applying the UGW method to damage detection of rod members also has been intensively studied. Shoji et al. experimentally studied the UGW-based detection of anchor rods embedded in soil, and successfully identified the artificial V-shaped necking defect [21,22]. Stepinski et al. proved that time-frequency analysis of UGWs could be used to assess the integrity of rock bolts [23]. Rong et al. analyzed the pulse echo of UGWs in rock bolts, and found that the wave mode L(0, 1) is more sensitive to the area of damage than other wave modes [24]. Zhao et al. found that the guided wave echoes of the corrosion pit in the low-frequency longitudinal wave mode are well correlated with the position and cross-sectional area of the pits [25]. Amjad et al. studied the time-of-flight (TOF) of low-frequency longitudinal mode UGWs in steel rods with various degrees of corrosion, and found that the corrosion level can be quantified by the change in TOF [26].

The influence of threads on the propagation characteristics of UGWs is theoretically insignificant, since the size of threads is much smaller than the wavelength of low-frequency UGWs [27,28]. Therefore, in the numerical simulation of UGWs propagation in threaded rods, especially for externally threaded rods (such as anchor rods [23,27] and rebars [26]), they are mostly simplified as ideal rods. However, for the real threaded rods, especially for the internally threaded rods, the threads cause a change in the cross-section and thus result in significant changes in echoes [29]. To the authors’ knowledge, few studies on UGW-based detection of internally threaded rods have been reported. Meanwhile, most of the previous studies on UGW-based damage detection of threaded rods focused on inspection of artificial damages by analyzing the linear characteristic variations of guided waves [21,22,23,24,25,26,27]. However, the first thread of the internally threaded rod is prone to fatigue due to stress concentration [30], while the linear characteristics of guided waves (e.g., wave speed, echo amplitude, etc.) are not sensitive to micro-fatigue cracks [15,31]. The detection of the fatigue cracks in the threaded rod using UGW-based method is still a technological challenge and worthy of being investigated. This paper carries out a study towards this challenge. 

The layout of the paper is as follows: Section 2 presents the propagation characteristics and nonlinear characteristics of guided waves in rods. In Section 3, numerical simulations are carried out to explore the feasibility of using guided waves to detect cracks in threaded rods. Section 4 gives an experimental validation of the UGW-based detection of fatigue cracks in threaded rods. The conclusions of the study are drawn in Section 5.

## 2. UGWs in Cylindrical Rod 

### 2.1. Propagation Characteristics 

**Figure 1:**
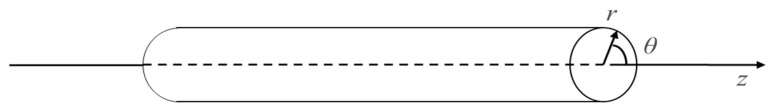
Infinitely long cylindrical rod with diameter of *r*.

The threaded rod can be simplified as a cylindrical rod considering that the size of thread is much smaller than the wavelength of the UGWs, and thus the UGWs in the threaded rod can be theoretically idealized as UGWs in the cylindrical rod [14,27,28]. Figure 1 presents an infinitely long cylinder with a radius of *r*, which is a homogeneous isotropic elastic medium, and its central axis coincides with the *z*-axis. There are three groups of modes for the UGWs propagating in the cylinder, i.e., longitudinal mode, torsional mode and flexural mode, which are labeled as L(0, m), T(0, m) and F(n, m), respectively, where n is the harmonic order and m is a sequence number. They are distinguished from each other by the vibration patterns of the particles.

**Figure 2:**
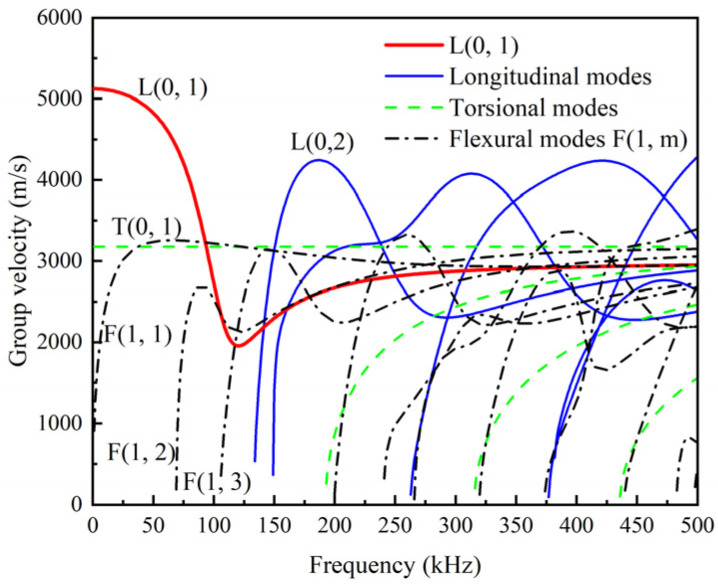
Dispersion curves of a typical infinitely long cylindrical rod with diameter of 27 mm.

The frequency equation of the UGWs in the cylindrical rod can be obtained by substituting different stress-displacement boundary conditions into the differential equation of motion and the wave equation, and then the modes of UGWs in the cylindrical rod can be determined by solving the frequency equation [32]. Substituting the sectional size and material properties, including diameter *d*, density ρ, elastic modulus E, Poisson’s ratio v, etc., into the frequency equation, the wavenumber *k* at different frequencies *f* can be obtained by semi-analytical finite element (SAFE) method [33]. Then, the dispersion curves of the group velocity ($c_{g}$) of UGWs in the cylindrical rod can be determined by the relationship that $c_{g}=\frac{d\left(2\pi f\right)}{dk}$. Taking the steel cylinder with a diameter of 27 mm as an example, the dispersion curves of it are obtained by substituting diameter *d* = 27 mm, density *ρ* = 7850 kg/m^3^, elastic modulus *E* = 206 GPa, and Poisson’s ratio *v* = 0.3 into the above procedures, as shown in Figure 2. It is clearly shown that the group velocity of each mode varies with frequency, i.e., there is frequency dispersion.

The L(0, 1) modal UGWs at low frequencies (0–150 kHz) are frequently used in the previous studies and the linear characteristic variation of UGWs, including the amplitude and the wave velocity, are used to inspect the damage of rod members [21,22,23,24,25,26,27]. It also can be observed in Figure 2 that, at a given frequency of 60 kHz, the L(0, 1) modal UGWs has a larger group velocity than the T(0, 1) modal UGWs and the F(1, 1) modal UGWs. This observation means that the L(0, 1) modal signal reaches the damaged position of the rod significantly earlier than the signals of other two modes, which is conducive to mode identification and long-distance detection. The cut-off frequency of L(0, 2) modal UGWs is 134 kHz.

**Figure 3:**
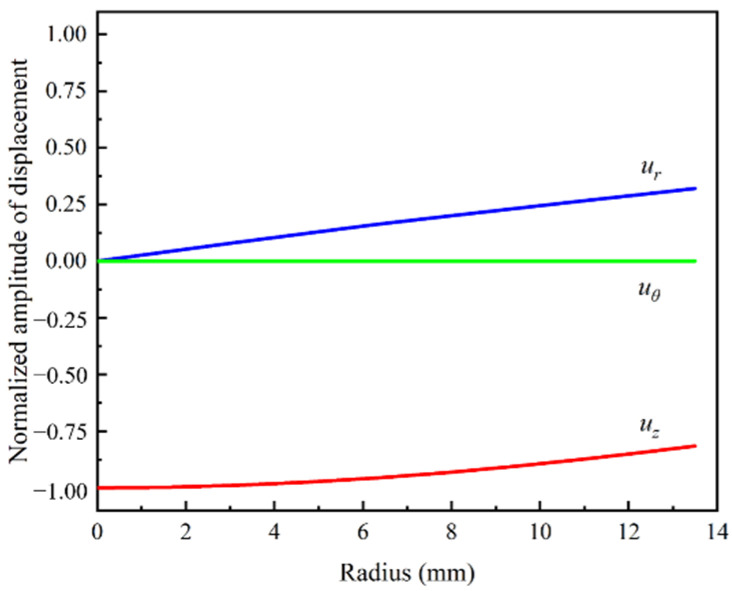
Displacement distribution of L(0, 1) modal UGWs in cylindrical rod at 60 kHz.

The displacement components of L(0, 1) modal UGWs at 60 kHz are plotted in Figure 3, which shows that there is no circumferential displacement *u_θ_* and the absolute value of the axial displacement *u_z_* is larger than the radial displacement *u_r_*. The displacement distribution of L(0, 1) modal UGWs in the cylindrical rod is simple, and its energy is concentrated in the axial direction. These features indicate that L(0, 1) modal UGWs are sensitive to circumferential defects and large radial (depth) defects, but not to axial defects. Besides, in cylindrical rods, the axisymmetric L modal UGWs is easier to be excited than the T modal UGWs and the asymmetric F modal UGWs [15,16]. Therefore, the L(0, 1) modal UGWs at low frequency (≤134 kHz) were chosen for damage detection of threaded rods.

The wavelength of the L(0, 1) modal UGWs at low frequencies is too large to detect tiny cracks effectively [34]. The linear characteristics of the low-frequency L(0, 1) modal UGWs are not sensitive enough for early warning of fatigue cracks [15,31]. Therefore, the nonlinear characteristics of the low-frequency L(0, 1) modal UGWs are also investigated and used for fatigue crack detection of threaded rods in this study.

### 2.2. Nonlinear Characteristics

#### 2.2.1. Nonlinear Parameters

When UGWs travel in a medium with fatigue cracks, higher order harmonic waves are generated due to Contact Acoustic Nonlinearity (CAN) [35]. For micro-cracks with rough surface, energy dissipation due to micro-contact and friction may also cause higher order harmonic waves [36].

The threaded rod is stressed by the contact forces between the threads and the connected components. The first thread, which is the transition between the smooth segment and the threaded segment, is prone to fatigue due to stress concentration. Local nonlinear damage occurs with the initiation and propagation of micro-cracks. In this case, the “CAN” can be simplified as the elastic modulus change at micro-crack $z_{1}$ [37], where the stress $\sigma_{1}$ is given by

(1) $$\sigma_{1}\left(z_{1}\right)=E_{1}\left(z_{1}\right)\varepsilon_{1}=\left(1-\beta max\left(\frac{\partial u\left(z_{1}\right)}{\partial z}\right)\right)E_{0}\frac{\partial u\left(z_{1}\right)}{\partial z}+\beta E_{0}{\left(\frac{\partial u\left(z_{1}\right)}{\partial z}\right)}^{2}$$

where $E_{0}$ is the initial elastic modulus; $E_{1}$ is the average value of the elastic modulus after the crack $z_{1}$ occurs; $u_{1}$ is the displacement at the micro-crack $z_{1}$; $\varepsilon_{1}$ is the strain at the micro-crack $z_{1}$; and β is the nonlinear parameter. The second term on the right side of Equation (1) is the second harmonic waves (SHW). In order to ensure the generation of SHW, the UGWs should oscillate and generate strain at the micro-crack. In other words, the inequation $\frac{\partial u\left(z_{1}\right)}{\partial z}\neq 0$ must be satisfied, which is also known as main mode vibration at the contact surfaces of micro-cracks.

Substituting Equation (1), $u_{1}=A_{1}^{L}\sin\left(kz_{1}-\omega t\right)$, $\max\left(\frac{\partial u\left(z_{1}\right)}{\partial z}\right)=A_{1}^{L}k\cos\left(kz_{1}-\omega t\right)$ and the solution of SHW $u_{1}^{H}=f\left(t\right)\sin\left(2kz_{1}-2\omega t\right)+g\left(t\right)\cos\left(2kz_{1}-2\omega t\right)$ into the wave equation and ignoring the third and higher order components, SHW can be further simplified as

(2) $$u_{1}^{H}=-\frac{\beta ck^{2}{\left(A_{1}^{L}\right)}^{2}t}{8}\cos\left(2kz_{1}-2\omega t\right)$$

where $\omega =2\pi f_{a}$, where $f_{a}$ is the central frequency of excitation signal; t is the time; and $\beta =\frac{A_{1}^{H}}{{\left(A_{1}^{L}\right)}^{2}}\frac{8}{k^{2}z}$ is usually referred as the acoustic nonlinear parameter, which can also be derived from the nonlinear Hooke’s law combined with the 1-D wave equation, representing the material nonlinearity [38].

In practice, the relative acoustic nonlinear parameter $\beta^{\prime }$ and the relative amplitude of the second harmonic $\beta_{re}^{\prime }$ given in Equations (3) and (4) are usually used to scale the acoustic nonlinearity.

(3) $$\beta^{\prime }=\frac{A_{1}^{H}}{{\left(A_{1}^{L}\right)}^{2}}$$

(4) $$\beta_{re}^{\prime }=\frac{A_{1}^{H}}{A_{1}^{L}}$$

where $\;A_{1}^{L}$ and $A_{1}^{H}$ are the amplitude of the fundamental guided waves and SHW in the frequency domain, respectively. The relative acoustic nonlinearity parameter $\beta^{\prime }$ is adopted to quantify the nonlinearity induced by local micro-cracks. The relative amplitude of the second harmonic $\beta_{re}^{\prime }$ is derived based on a bi-linear stiffness model [39], which considers the change of elastic modulus under different pressure and tension. Compared to $\beta_{re}^{\prime }$, $\beta^{\prime }$ amplifies the amplitude of variation at the fundamental frequency.

#### 2.2.2. Central Frequency of Excitation Signal

Considering that there may be only one end face of the threaded rod suitable for the coupling of the probe, the pulse-echo method is adopted to excite and receive low-frequency UGW signals. In this case, the L(0, 1) mode is the main mode of UGWs propagating in the threaded rod and the displacement of UGWs is mainly distributed in the axial direction. The fatigue crack at the root of the thread mainly grows in both radial and circumferential directions. In other words, the contact surface of micro-crack is perpendicular to the axial direction, which means the L-modal UGWs will cause opening and closing vibration of micro-crack. 

Noting that the cut-off frequency of the L(0, 2) mode is 134 kHz, when the second harmonic frequency ($2f_{a}$) is greater than 134 kHz, the L(0, 2) mode may be generated. In order to reduce the influence of other modes, the maximum excitation frequency is set as 65 kHz. Further, considering that too small excitation frequency will reduce the sensitivity of the signal to micro-cracks and will cause a long detection blind zone near the excitation end, the range for the central frequency of excitation is finally set at 30–65 kHz.

## 3. Numerical Study

### 3.1. UGWs Propagation in Undamaged Threaded Rod

#### 3.1.1. Finite Element Model

**Figure 4:**
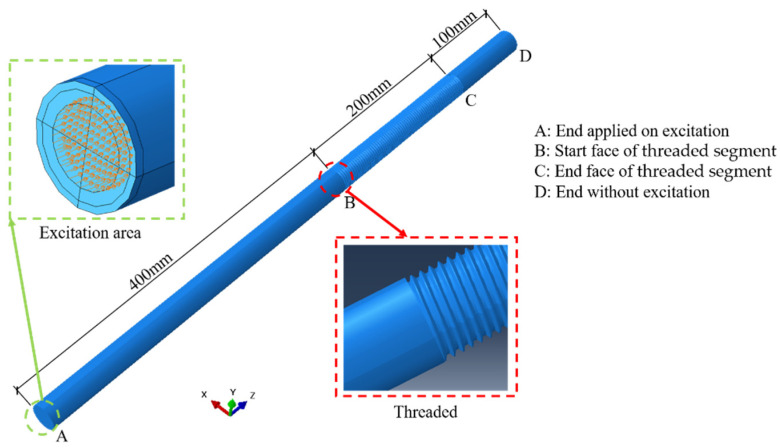
Numerical model of threaded rod.

A 700 mm-long threaded steel rod with a diameter of 27 mm is considered in this section. There is a 200 mm threaded segment between point *B* and *C*, as shown in Figure 4. The pitch of the threads is 3 mm and the depth of the threads is 1.62 mm. The material properties of the steel rod are the same as those given in Section 2.

The commercial finite element software Abaqus [40] was used to carry out the simulation. The mesh size Δx is suggested to be not larger than $\frac{\lambda }{20}$, where $\lambda =\frac{2\pi }{k}$ is the wavelength of the guided wave, to capture the fluctuation effect between the elements and then to ensure the accuracy and the convergence of the numerical computations [41,42]. Since larger frequency corresponds to smaller wavelength and central frequencies not larger than 70 kHz will be used, the wavelength λ = 70.1 mm corresponding to the central frequency $f_{a}=70$ kHz is used to determine the upper bound *λ*/20 = 3.5 mm. So, the global element size of the numerical model was set as Δx=2  mm and the model was meshed with eight-node brick elements (C3D8R) through adaptive meshing.

To simulate the pulse-echo excitation, an axial displacement excitation was applied on a circle area with a diameter of 18 mm at end A of the threaded rod (Figure 4), and a node 2 mm away from the end was selected as the receiver. In this way, there would only be axial and radial displacements in the rod, but no circumferential displacements. In other words, only the L(0, 1) modal guided waves would be excited.

**Figure 5:**
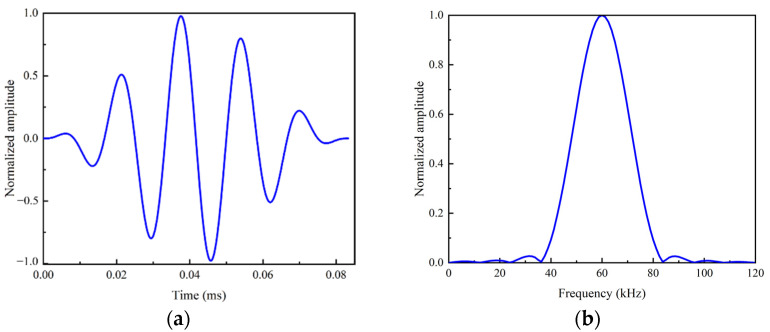
Excitation signal with central frequency of 60 kHz. (**a**) Excitation signal in time domain. (**b**) Excitation signal in frequency domain.

The sinusoidal waves with Hanning window at the central frequencies $f_{a}$ of 30 kHz, 40 kHz, 50 kHz, 60 kHz and 70 kHz were input as the excitation, respectively. Here the maximum excitation frequency is set as 70 kHz to check the influence of L(0, 2) mode to the nonlinear effect of guided waves. The excitation signal is expressed as follows:

(5) $$f\left(t\right)=\{\begin{array}{c} A((1-\cos\left(\frac{2\pi f_{a}t}{n}\right))\sin\left(2\pi f_{a}t\right)\\ 0 \end{array}\begin{array}{c} 0<t<\tau \\ t>\tau \end{array}$$

where $\tau =\frac{n}{f_{a}}$ is the excitation time; n is the number of modulation cycle; and A is the amplitude of the signal. Here *n* = 5 and A=0.5 were used. With the increasing of n, the energy leakage of the spectrum decrease, which means that more energy is concentrated to central frequency $f_{a}$. Meanwhile, the time-history signal is more complex due to the increase in the excitation time *τ*. Figure 5 shows a typical normalized excitation signal with the central frequency $f_{a}=60\;\mathrm{kHz}$, whose energy is concentrated to the central frequency $f_{a}$, which is beneficial to reduce the dispersion phenomenon of the guided wave. Each signal propagated in the rod for 0.0032 s and this process was tracked by the Explicit Solver embedded in Abaqus.

The guided wave signals used in this paper are all normalized as follows:

(6) $$\bar{a}\left(t\right)=\frac{a\left(t\right)-\mu }{\sigma }\;\mu =\frac{1}{N}\overset{N}{\underset{i=1}{\sum }}a\left(t_{i}\right)\;\sigma^{2}=\frac{1}{N-1}\overset{N}{\underset{i=1}{\sum }}{\left(a\left(t_{i}\right)-\mu \right)}^{2}$$

where a(t) is the guided wave time-history signal; μ is the mean of a(t); σ is the mean square error; and  N is the number of sampling points. After the normalization, the mean amplitude of the signals becomes zero and the mean square error becomes one. 

#### 3.1.2. Signal Analysis

**Figure 6:**
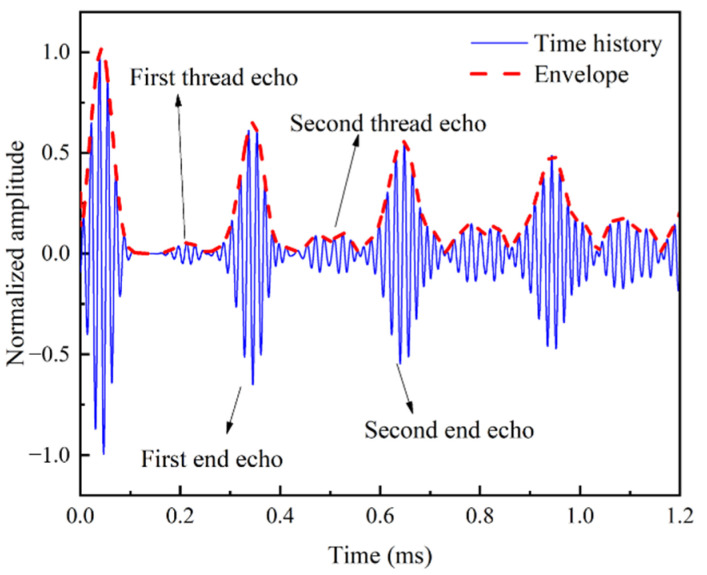
Time history and envelope of UGWs with central frequency of 60 kHz propagating in threaded rod.

As shown in Figure 6, thread-induced, cross-sectional changes cause some signals to be reflected and these reflected signals are called thread echo. With the increasing of the propagation time, the thread echo is enhanced by multiple reflections at threaded cross-sections. As a result, the waveform becomes more complex and the end echo gradually decreases due to energy loss. 

**Figure 7:**
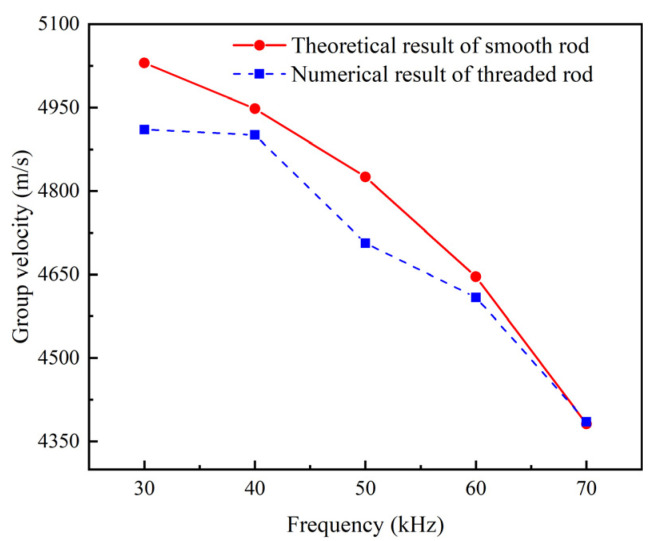
Group velocity under different excitation frequencies.

**Figure 8:**
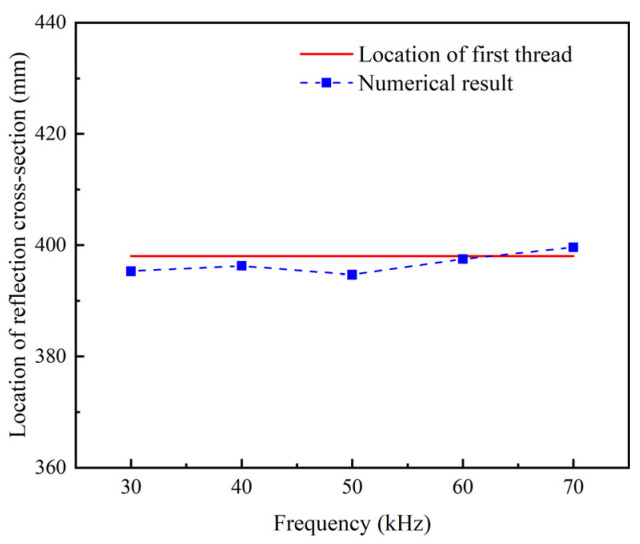
Location of refection cross-section under different excitation frequencies.

**Figure 9:**
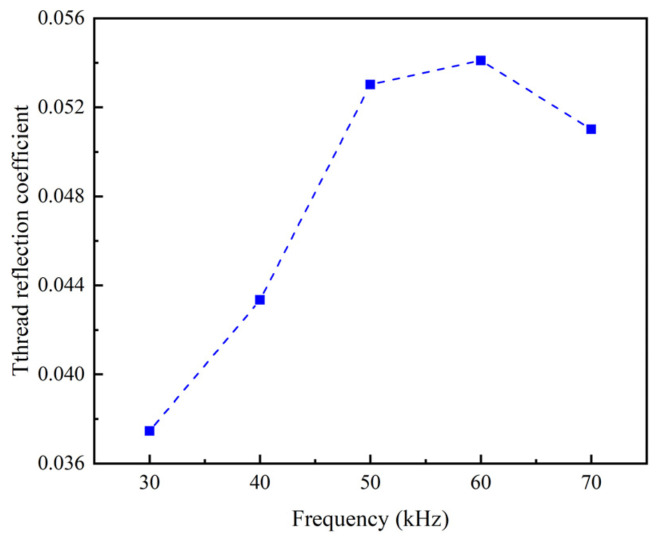
Thread reflection coefficients at different excitation frequencies.

The group velocity $c_{g}$, the thread reflection coefficient $R_{f}$, and the location of reflection cross-section $Loc_{f}$ as defined in Equations (7)–(9) can be determined by picking up the excitation signal, the thread echo, and the peak amplitude of the end echo and their propagation times.

(7) $$c_{g}=\frac{2L}{t_{1}-t_{0}}$$

(8) $$R_{f}=\frac{H_{f}}{H_{0}}$$

(9) $$L{\mathrm{oc}}_{f}=\frac{c_{g}}{2}\left(t_{f}-t_{0}\right)$$

where $H_{0}$ and $H_{f}$ are the peak amplitudes of the excitation signal and the thread echo, respectively; and $t_{0}$, $t_{f}$ and $t_{1}$ are the propagation times corresponding to the peak of the excitation signal, the thread echo and the end echo, respectively. Here the time history signal of the first round-trip is selected to calculate the three parameters defined in Equations (7)–(9). The obtained results corresponding to different excitation frequencies are plotted in Figure 7, Figure 8 and Figure 9.

It can be seen from Figure 7 that, in the given frequencies, the group velocity obtained by the numerical simulation of the threaded rod is close to the theoretical result of the simplified smooth rod. To some degree, it justifies the simplification that a threaded rod can be treated as a smooth rod with the same diameter in the analytically solving of the dispersion curve.

It is found that the location of the reflection cross-section is close to the location of the first thread (Figure 8). It indicates that the interface between the smooth segment and the threaded segment is the main cause of the thread echo.

Figure 9 shows that the thread reflection coefficient reaches maximum at 60 kHz. It is also found that the location of reflection cross-section is closest to the location of the first thread at 60 kHz (Figure 8). These observations indicate that the UGWs at 60 kHz is most sensitive to the cross-sectional change at the first thread.

**Figure 10:**
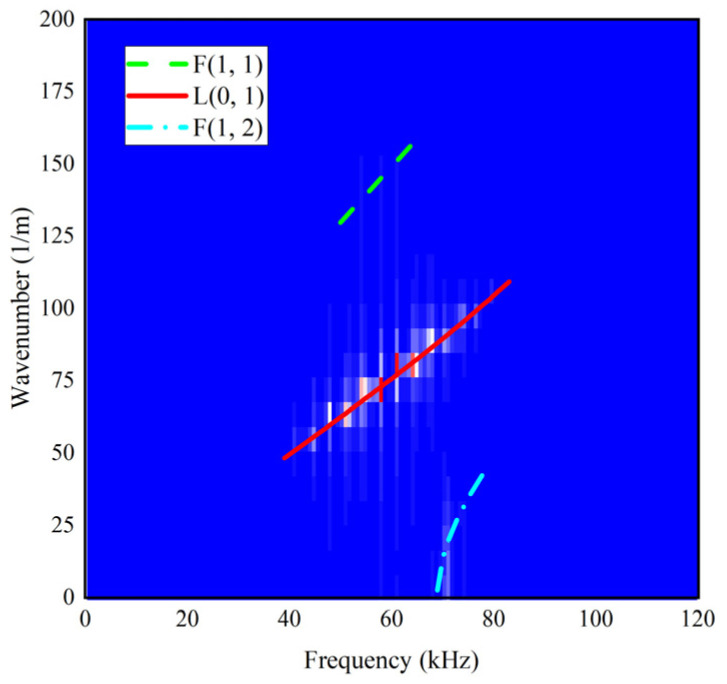
Wavenumber diagram based on 2D Fourier transform at 60 kHz.

To further investigate the effect of the threads to the propagation of the UGWs, the frequency versus wavenumber relationship obtained by applying 2D Fourier transform on the axial displacement signals of a set of nodes along the length of the rod [43] is plotted in Figure 10. It shows that the wavenumber of the signal propagating in the threaded rod coincides with the wavenumber of L(0, 1) modal UGWs, which indicates that the L(0, 1) mode is the main mode of the signal traveling in the rod. It also shows that there are slight non-axisymmetric bending modes F(1, 1) and F(1, 2) because of the non-axisymmetric thread segment.

### 3.2. UGWs Propagation in Threaded Rod with Crack

#### 3.2.1. Modeling of “Breathing” Crack

To mimic the fatigue crack, the “surface-to-surface contact” interaction in Abaqus was applied to each surface of the crack, and the normal behavior and the tangential friction coefficient of the interaction were set as “hard” contact and 0.1, respectively.

**Figure 11:**
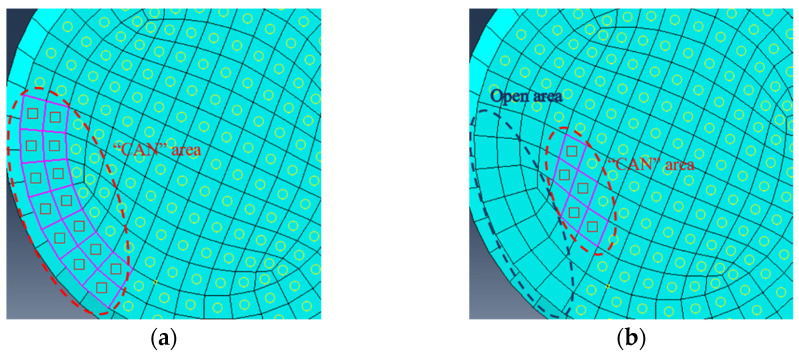
Modeling of “breathing” crack. (**a**) “CAN” area of fully “breathing” crack. (**b**) “CAN” area of partially “breathing” crack.

There are 6 cases of “CAN” area considered to mimic fatigue cracks with different radial depths. They are named as C1-C5 and CB6, and the corresponding “CAN” areas are given in Table 1. Among them, the cracks in cases C1-C5 are fully “breathing” and the whole cracked area has “CAN”. The crack in case CB6 is deemed as open wider than others and there is no “breathing” effect at the partial area close to the surface. Note, that the cases C5 and CB6 have the same cracked area. Illustrations for the numerical modeling of fully “breathing” crack and partially “breathing” crack are shown in Figure 11. 

#### 3.2.2. Signal Analysis

The time domain envelopes of full signals are obtained by normalizing and Hilbert transforming the guided wave signals in different cases successively. The Hilbert transform was performed to the difference of normalized guided wave signals under undamaged state and damaged state to obtain time domain envelopes of residual signals.

**Figure 12:**
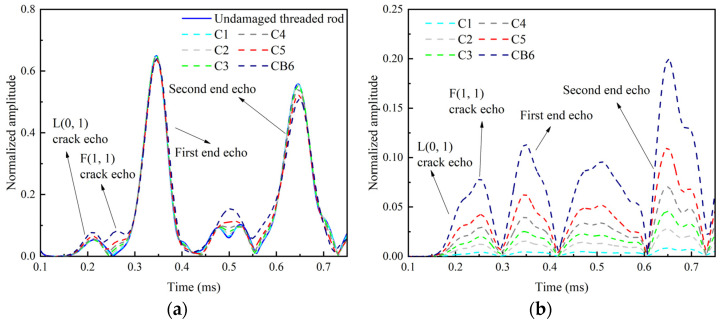
Time domain envelopes of signals at 60 kHz. (**a**) Time domain envelopes of full signals. (**b**) Time domain envelopes of residual signals.

As shown in Figure 12, the amplitude of the crack echo increases and the amplitude of the end echo decreases with the increase in the crack area. It is notable that there are two obvious wave packets before the first end echo. The first wave packet is the L(0, 1) modal crack echo, and the second one is the F(1, 1) modal echo, which is generated by the guided wave conversion. Although with the same crack area as case C5, the amplitude of the crack echo of CB6 is larger than that of C5 and the amplitude of the end echo of CB6 is smaller than that of C5. This finding indicates that the energy reflected by the full “breathing” crack is smaller than that reflected by the partial “breathing” crack and more energy transmits through the full “breathing” crack than the partial “breathing” crack.

Again, the first round-trip echo in time domain is selected to calculate the linear parameters including the group velocity difference $\Delta c_{g}\;$ of the L(0, 1) mode between the damaged state and undamaged state, the reflection coefficient of the crack $R_{f}$, and the location of the crack $Loc_{f}$.

**Figure 13:**
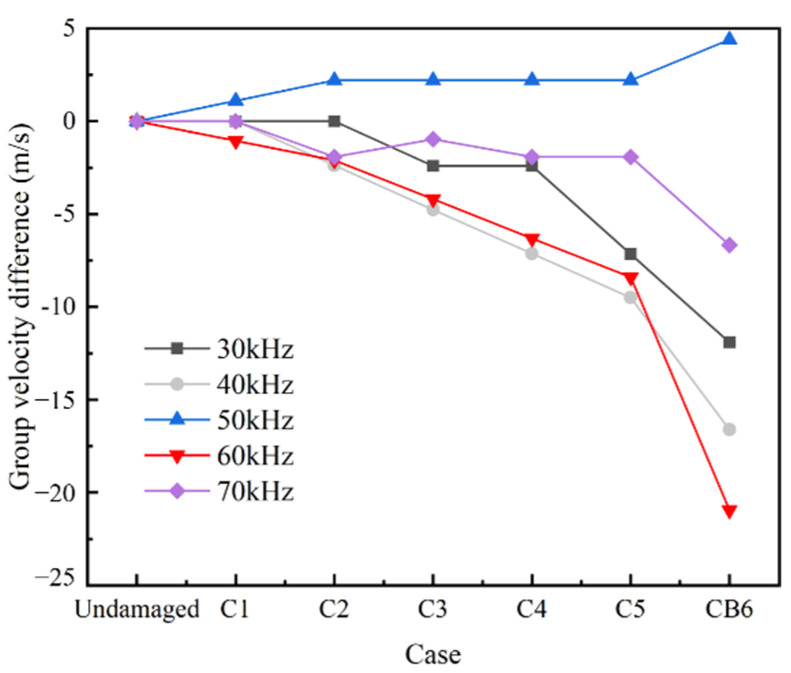
L(0, 1) mode group velocity difference.

**Figure 14:**
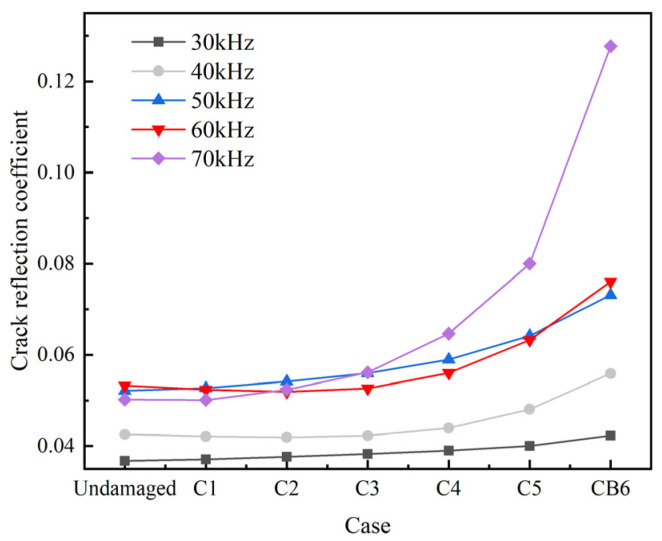
Crack reflection coefficient diagram.

**Figure 15:**
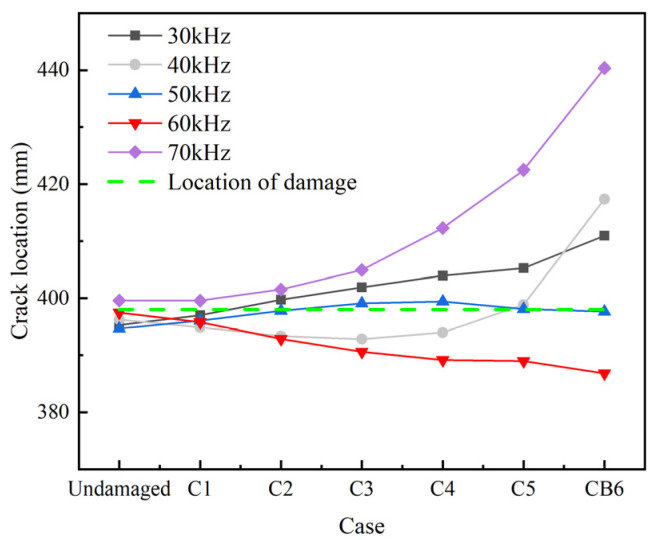
Crack location.

As shown in Figure 13, the group velocities of L(0, 1) modal UGWs in the damaged rod is lower than that in the undamaged rod at central exciting frequency of 30–70 kHz except for 50 KHz. As the crack area increases, the absolute differences between the group velocities of the damaged rod and the undamaged rod gradually increases. The reflection coefficient of the crack at different central exciting frequencies generally increases as the crack area increases (Figure 14). It is also worth noting that the reflection coefficient at 30 kHz and 40 kHz increases very slowly as the crack area increases (Figure 14), which indicates that UGWs at these frequencies are not sensitive to cracks. When the central exciting frequency is over 50 kHz, the reflection coefficient increases significantly after case C4 (Figure 14). Figure 15 shows that the UGWs at 50 kHz are more suitable for damage localization than the others.

The sum of residual square of the main frequency signal in time domain *ARS*, as expressed by Equation (10), is an important index for the change of signal in time domain due to damage. It is the accumulated square of the difference between the amplitudes of time-history signals in undamaged state and damaged state.

(10) $$ARS=\sum {\tilde{a}}^{2}=\overset{t=TIME}{\underset{t=0}{\sum }}{\left(a_{Base,t}-a_{Dam,t}\right)}^{2}{}^{}$$

where $a_{Base,t}$ is the amplitude of the signal in the undamaged component at the central frequency at time *t*; and $a_{Dam,t}$ is the amplitude of the signal in the damaged component at central frequency at time *t*.

**Figure 16:**
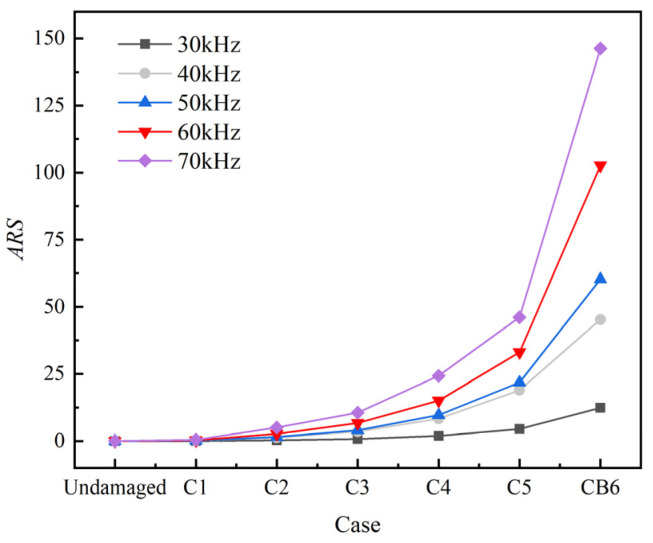
Sum of residual square of main frequency signal in time domain.

As shown in Figure 16, the *ARS* increases with the increasing of the crack area and the central frequency. In particular, the *ARS* increases very slightly with the increasing of the crack area at 30 kHz, while it increases much significantly with the increasing of the crack area at frequencies above 30 kHz, especially from the case C4 to CB6. Besides, the CB6 has a higher *ARS* than the C5 under the same conditions. 

**Figure 17:**
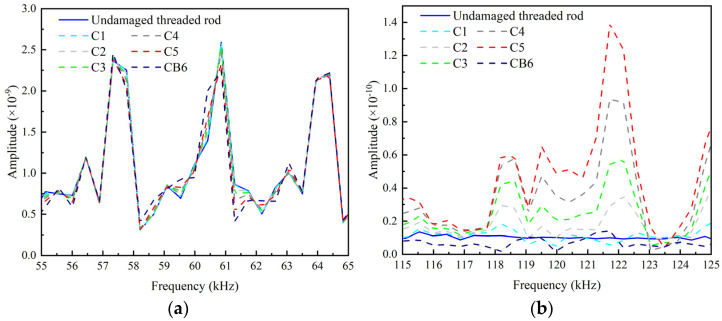
Spectrum at central exciting frequency of 60 kHz. (**a**) Main frequency spectrum. (**b**) Second-order frequency spectrum.

To explore the influence of the change of the “CAN” area on the nonlinear characteristics of the UGW, the short-time Fourier transform was performed to the signals and the result was shown in Figure 17. 

It can be found that with the increasing of the crack area, the amplitude of the main frequency spectrum slightly decreases. On the contrary, the amplitude of the second order frequency increases and the SHW is significantly enhanced by the increasement of the crack area. It is noteworthy that the second-order frequency curve of CB6 is close to that of the undamaged case. This indicates that with the appearance of separated area, the nonlinearity of the crack decreases and thus the SHW reduces.

**Figure 18:**
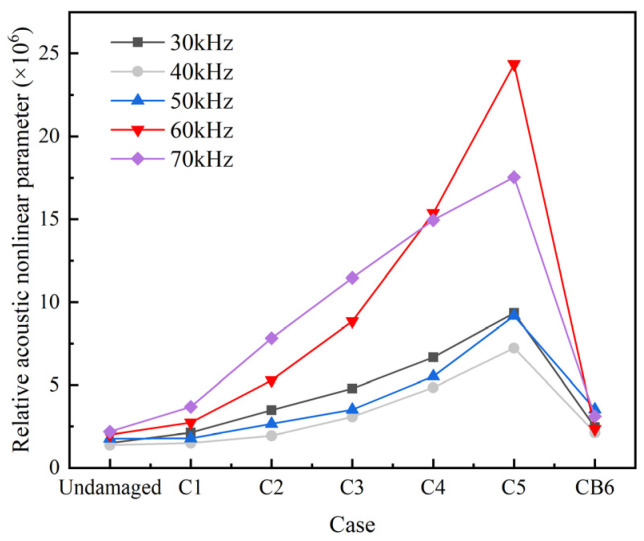
Relative acoustic nonlinear parameter $\beta^{\prime }$ at different cases.

**Figure 19:**
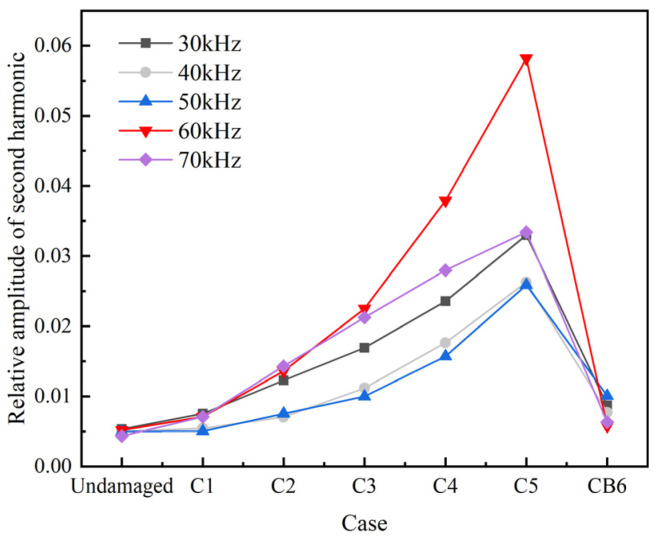
Relative amplitude of second harmonic $\beta_{re}^{\prime }$ at different cases.

The peak amplitude in the central frequency $f_{a}$ band ($f_{a}-$5 kHz, $f_{a}+$5 kHz) and in the second-order frequency band (2$f_{a}-$5 kHz, 2$f_{a}+$5 kHz) are denoted by $\;A_{1}^{L}$ and $\;A_{1}^{H}$, respectively. The relative acoustic nonlinear parameter $\beta^{\prime }$ and the relative amplitude of the second harmonic $\beta_{re}^{\prime }$ are obtained using Equations (3) and (4), as shown in Figure 18 and Figure 19.

Figure 18 and Figure 19 show that, within the given range of central exciting frequencies, the nonlinear parameters $\beta^{\prime }$ and $\beta_{re}^{\prime }$ obviously increase from the undamaged case to C5 with the increasing of the nonlinear damage area of “CAN”. This finding indicates that the nonlinear parameters can be used to detect the nonlinear damage. However, for the case CB6, which has a same area of “CAN” to C5, the nonlinear indexes drop to a level close to the case C1. The observation reveals that the nonlinear indexes characterized the magnitude of the area of “CAN”, but not the whole area of crack. When $f_{a}=70$ kHz, the amplitudes of $\beta_{re}^{\prime }$ and $\beta^{\prime }$ are smaller than those at 60 kHz except for $\beta^{\prime }$ in cases C1-C3, which shows that in general the existence of L(0, 2) mode reduce the nonlinear effect of guided waves traveling in rods. It is also worth noting that the nonlinear indexes $\beta_{re}^{\prime }$ and $\beta^{\prime }$ are more sensitive to the change of the “CAN” crack area at 60 kHz than at other frequencies.

According to the above numerical study, it can be found that when the central frequency of the excitation is 60 kHz, both the linear characteristics of UGWs, such as the reflection coefficient $R_{f}$ of the main frequency signals and the accumulated value ARS of the square residual of the main frequency signals, and the nonlinear characteristics of UGWs, such as the relative amplitude of the second harmonic $\beta_{re}^{\prime }$ and the relative acoustic nonlinear parameter $\beta^{\prime }$, obviously vary with the change of the crack area. Therefore, the central frequency of excitation signal is set as 60 kHz in the following experimental study. 

## 4. Experiment

### 4.1. Specimen and Setup

**Figure 20:**
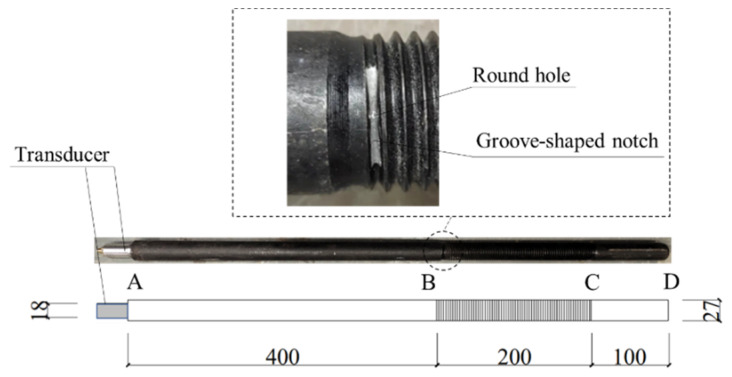
Geometry of specimen.

A specimen, the same as the threaded rod considered in the numerical study, was used in the experiment. The detailed geometry of it is shown in Figure 20. A groove-shaped notch with a length of about 13 mm, a width of about 1 mm, and a depth of about 2 mm was machined at the interface between the smooth segment and the threaded segment (Figure 20). A round hole with a diameter of about 1 mm and a depth of about 0.5 mm was machined in the middle of the groove notch. The groove notch and round hole were used as initial defects to initiate and accelerate the formation and growth of cracks.

**Figure 21:**
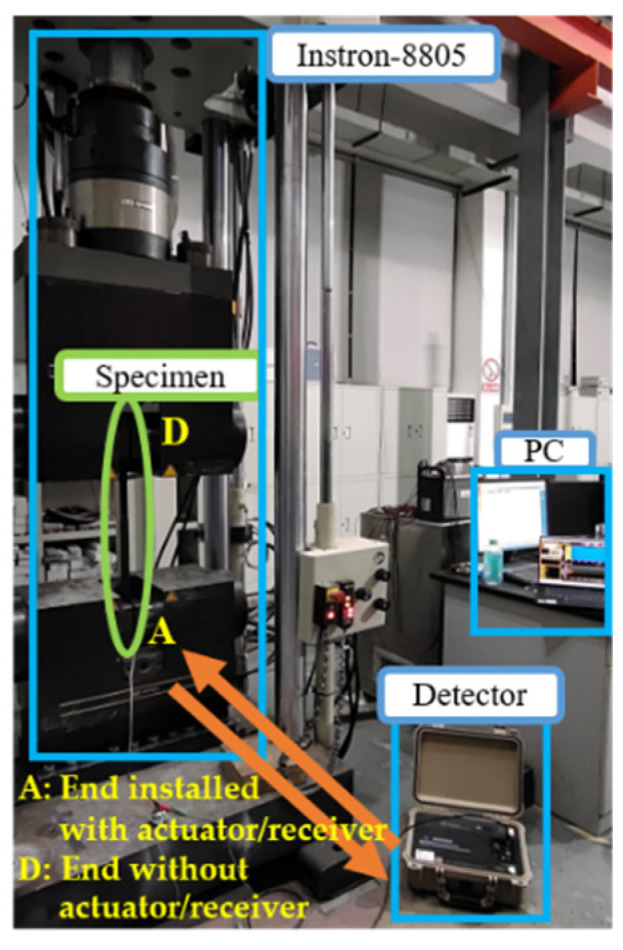
Photograph of experimental setup.

The experimental setup is shown in Figure 21. The Instron8805 test machine was used to carry out the constant-amplitude tensile fatigue testing. A length of 90 cm of each end of the specimen was clamped by the test machine with a clamping force of 4.5 MPa. A piezoelectric probe, which works as both actuator and receiver in the plus-echo mode, was bonded at the end A of the threaded rod by using Loctite EA E-05CL adhesive. The piezoelectric probe was connected to the MSGW30 ultrasonic guided wave detector [44] through a data cable, and the detector was connected to the PC via WIFI.

A periodic sinusoidal tensile force whose maximum and minimum are 131.0 kN and 39.3 kN, respectively, was applied to the specimen by the test machine at a frequency of 8 Hz. This cyclic load caused a stress periodically varying from 68.6 MPa to 228.6 MPa in the specimen. After every 20 k (20,000) cycles of loading, the specimen was unloaded. Then sinusoidal waves with 3-cycle and 10-cycle Hanning windows at a central exciting frequency of 60 kHz were input as excitation signals in sequence. For each excitation signal, 20 echo signals were collected by the transducer at a sampling frequency of 2 MHz. Each sampling lasted for 0.004 s. During the experiment, the environment temperature was kept constant at 13 °C. Besides, a total of 80 initial signals were collected as baseline signals before cyclic loading. 

### 4.2. Fatigue Crack Development

**Figure 22:**
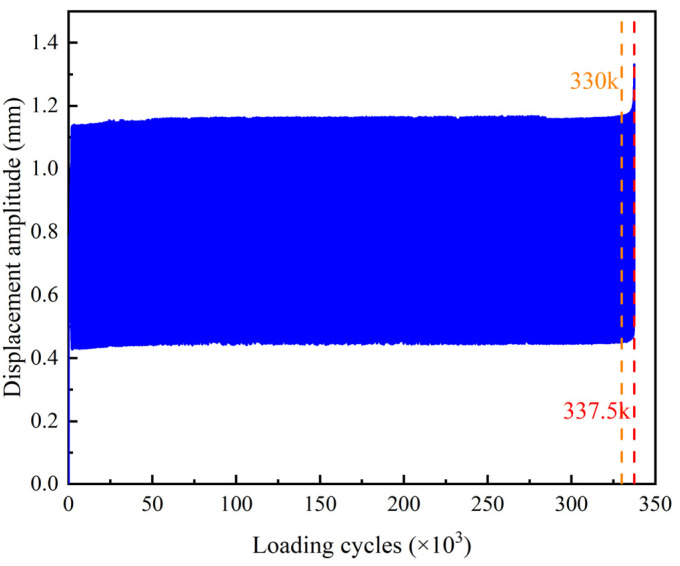
Displacement of upper clamp of test machine.

The vertical displacement of the upper clamp of the test machine throughout the experiment is shown in Figure 22. It shows that the amplitude of the displacement keeps constant before 330 k cycles of loading and then obviously rises till to 337.5 k cycles when the rod abruptly breaks at the cross-section with initial defect due to rapid growth of a fatigue crack.

**Figure 23:**
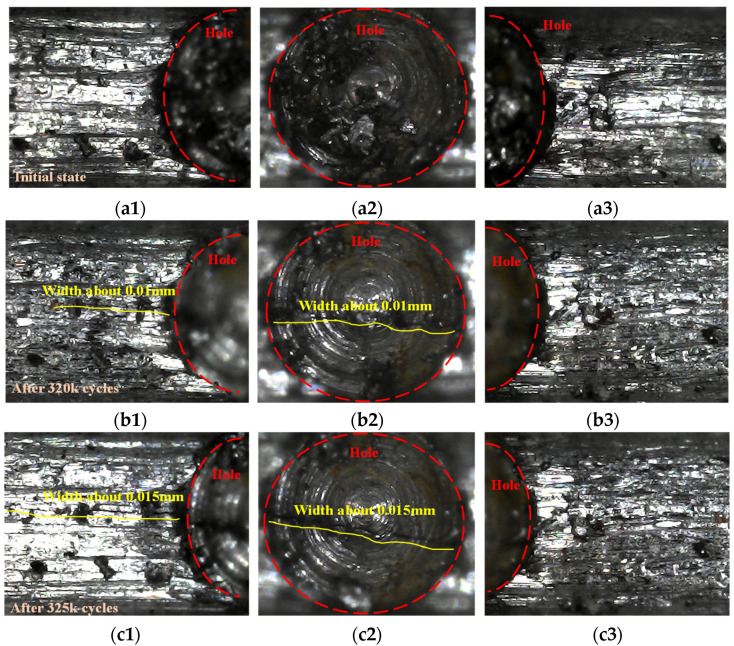

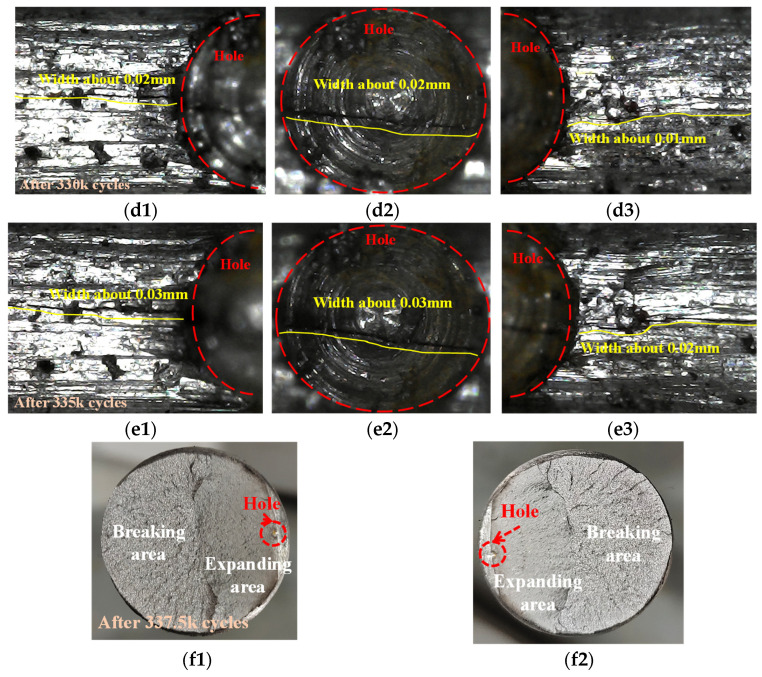
Crack growth and broken at defected cross-section. (**a1**) left side of hole; (**a2**) hole bottom; (**a3**) right side of hole; (**b1**) left side of hole; (**b2**) hole bottom; (**b3**) right side of hole; (**c1**) left side of hole; (**c2**) hole bottom; (**c3**) right side of hole; (**d1**) left side of hole; (**d2**) hole bottom; (**d3**) right side of hole; (**e1**) left side of hole; (**e2**) hole bottom; (**e3**) right side of hole; (**f1**) Upper broken surface (close to end D); (**f2**) Lower broken surface (close to end A).

The development of the fatigue crack during the cyclic load is shown in Figure 23. At the initial state, there was no visible crack around the initial defect (Figure 23a). After 320 k cycles of loading, a tiny crack through the artificial hole was observed (Figure 23b) and rapidly expanded (Figure 23c–e). The rod was broken after 337.5 k cycles of loading. The broken section was perpendicular to the longitudinal axis of the rod and crack expanding area and sudden breaking area can be recognized on the broken section (Figure 23f).

### 4.3. Experimental Results

**Figure 24:**
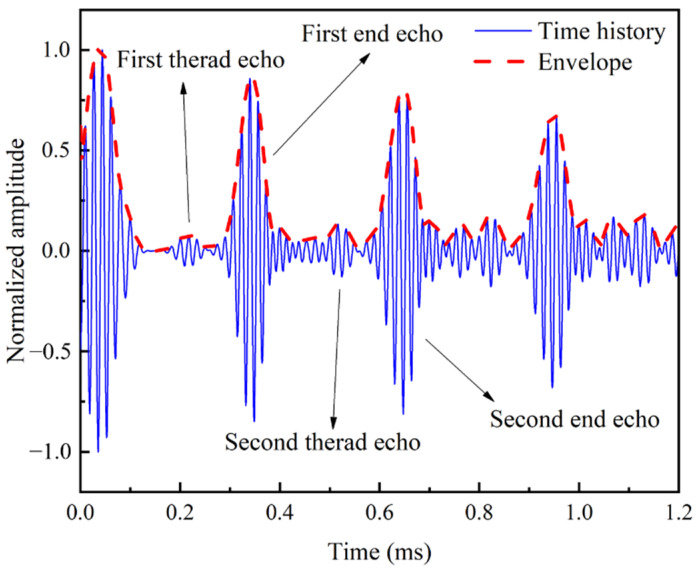
Filtered echo signal at initial baseline state.

In order to further remove the random noise, the guided wave signals obtained in the experiment are filtered by the continuous wavelet transform [45] and then normalized according to Equation (6). Figure 24 illustrates the filtered echo signal collected by the piezoelectric probe before the loading test, when sinusoidal waves with a 3-cycle Hanning window at 60 kHz are used as the excitation signal.

The reflection coefficient $R_{f}$ of the baseline signal is 0.078. The group velocity of the UGWs determined based on the baseline signal is 4548 m/s which is 2.1% smaller than the theoretically prediction and 1.4% smaller than the numerically prediction of the group velocity of the L(0, 1) modal UGWs. The geometric and material deviations of the specimen and the random vibrations of the bonding layer might cause the difference [46,47]. This general agreement in the group velocity verifies that the L(0, 1) mode is the main mode of the baseline signal.

**Figure 25:**
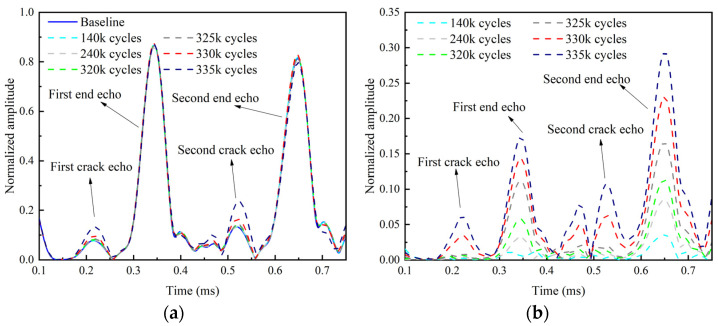
Envelopes of fundamental and residual echo signals after various of loading cycles. (**a**) Envelope of echo signal; (**b**) Envelope of residual echo signal.

Figure 25 shows the envelopes of the filtered fundamental echo signal and residual echo signal after various loading cycles. It is found that the amplitudes of the crack echo and its residual increase rapidly after 325 k cycles, while those of the end echo decreases rapidly. It is also found that the second echo changes more significantly with the increasing of load cycles than the first echo (Figure 25). 

**Figure 26:**
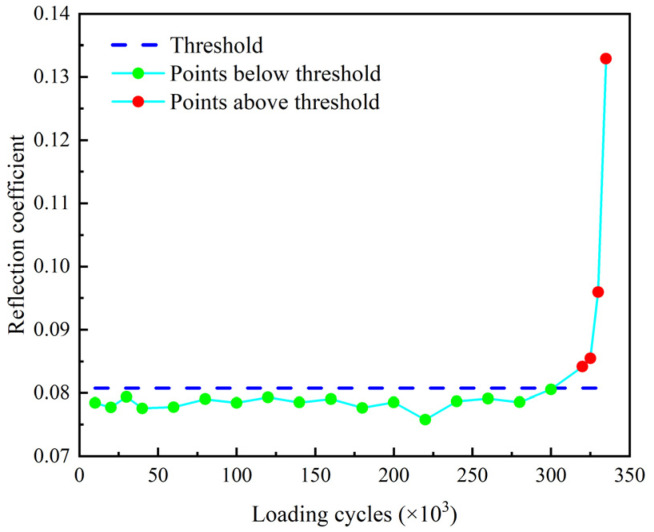
Reflection coefficient $R_{f}$ of filtered echo signal.

**Figure 27:**
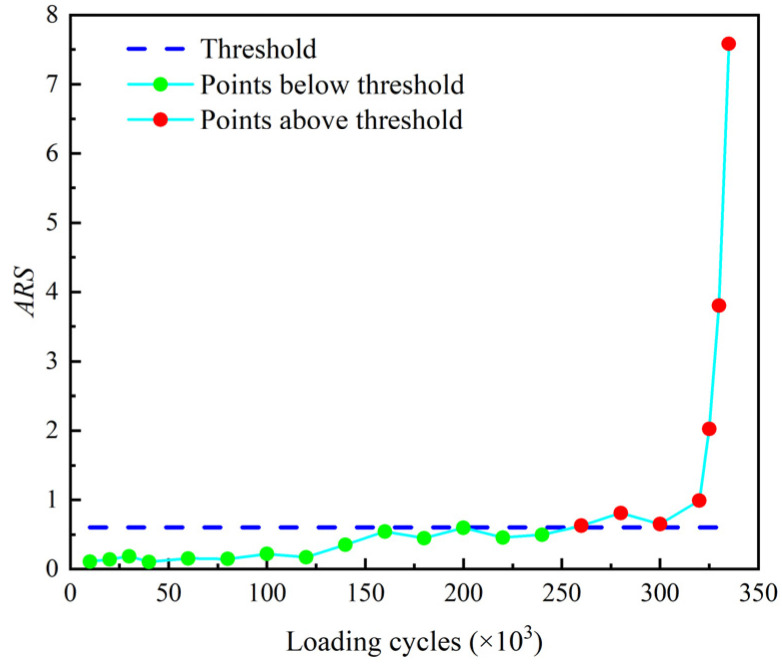
Accumulative residual squares *ARS* of filtered echo signal.

The reflection coefficient $R_{f}$ and the accumulative residual squares *ARS* of the echo signals are determined and plotted in Figure 26 and Figure 27 where the threshold for crack identification is set as the mean of the corresponding parameter determined from the baseline signals plus three times of the standard deviation. 

It shows that both the reflection coefficient $R_{f}$ and the accumulative residual squares *ARS* exceed the threshold and monotonically increase after 320 k loading cycles. This verifies that both the reflection coefficient $R_{f}$ and the accumulative residual squares *ARS* are effective indexes for damage detection of the threaded rod. Moreover, the *ARS* slightly exceeds the threshold after 260 k loading cycles when there is no crack visually observed in the experiment. This indicates that the *ARS* may be used as an early warning index for the fatigue crack of threaded rods.

**Figure 28:**
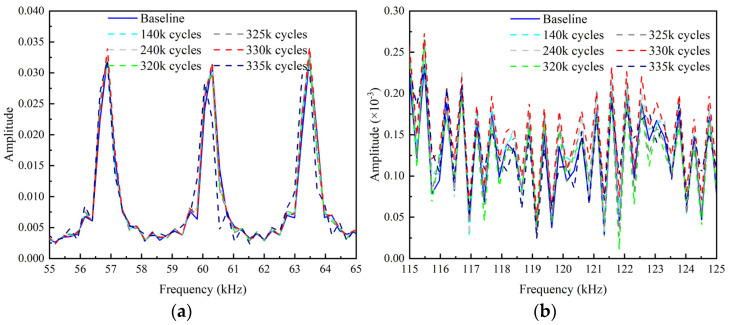
Frequency spectrums after various loading cycles. (**a**) Fundamental frequency spectrums around central frequency; (**b**) Second harmonic frequency spectrums around double of central frequency.

The frequency spectrums obtained by performing short-time Fourier transform on the echo signals are shown in Figure 28. In the early loading stage, there is no significant difference in the frequency spectrum between the test and baseline signal. After 335 k loading cycles, the amplitudes in the fundamental frequency band begin to shift and decrease obviously. The peaks in the second harmonic frequency band increase as the loading cycles increase. However, after 335 k loading cycles, the peaks of the second harmonic frequency spectrum decreases compared to that after 330 k loading cycles. The reason for this phenomenon is that the wider opening of the crack after 335 k loading cycles reduces the “CAN” area, which is similar to the case CB6 in the numerical study.

**Figure 29:**
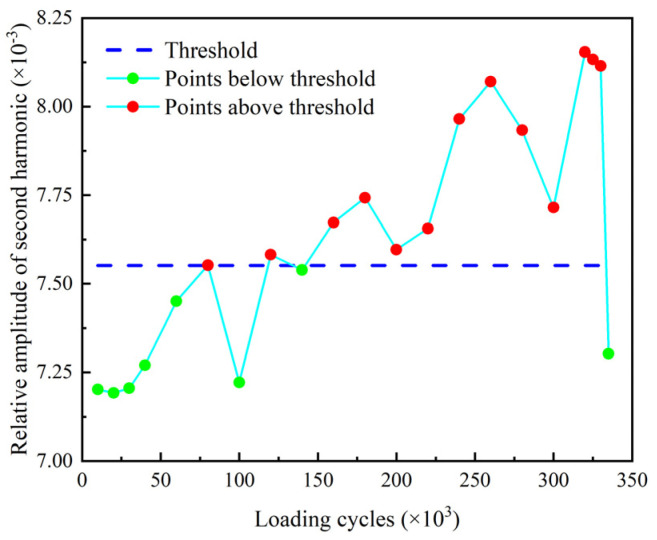
Relative amplitude of second harmonic $\beta_{re}^{\prime }$ versus loading cycles.

**Figure 30:**
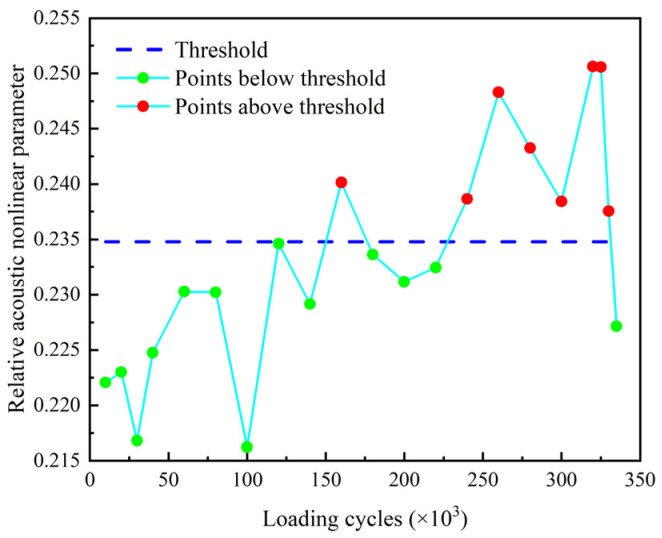
Relative acoustic nonlinear parameter $\beta^{\prime }$ versus loading cycles.

The relative amplitude of the second harmonic $\beta_{re}^{\prime }$ and the relative acoustic nonlinear parameter $\beta^{\prime }$ of the second harmonic are shown in Figure 29 and Figure 30. The thresholds for these two indexes are also defined as the mean of the corresponding parameter determined from the baseline signals plus three times of the standard deviation. It can be found that $\beta_{re}^{\prime }$ and $\beta^{\prime }$ stably exceed their thresholds after 160 k loading cycles and 240 k loading cycles, respectively. Then, they are fluctuating upward and reach their peaks at 320 k loading cycles. After that, they continuously decrease as the number of loading cycles increases, and fall below the threshold at 335 k loading cycles. It can be seen that the nonlinear indexes $\beta_{re}^{\prime }$ and $\beta^{\prime }$ are able to warn the damage much earlier than the linear indexes $R_{f}$ and *ARS*.

### 4.4. Influence of Number of Modulation Cycles 

To investigate the influence of the number of modulation cycles on the echo signals, sinusoidal waves with 10-cycle Hanning window at 60 kHz was also used as excitation signal. With the increasing modulation cycles of the excitation signals, the spectral energy is more concentrated and the bandwidth is smaller, which is conducive to the capture of nonlinear characteristics based on the spectrum. However, it also means longer duration of the excitation signal, causing the echo signals to be overlapped by the excitation signals, which makes it is difficult to obtain linear characteristics. Therefore, only the nonlinear characteristics are investigated in this chapter. 

**Figure 31:**
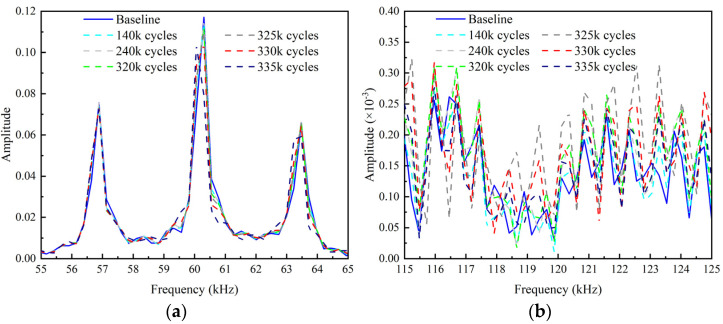
Frequency spectrums after various loading cycles corresponding to 10-cycle excitation. (**a**) Fundamental frequency spectrums around central frequency; (**b**) Second-order frequency spectrums around double of central frequency.

The frequency spectrums corresponding to the 10-cycle excitation signal are shown in Figure 31. Comparing Figure 31a with Figure 28a, it can be noted that, with the increment of the number of modulation cycles, the energy of UGWs is more concentrated to the central frequency, the amplitudes of the fundamental spectrums increase greatly, and the frequency shift becomes more obvious. Comparing Figure 31b with Figure 28b, it can be found that the amplitudes of the second harmonic frequency spectrums also increase, but not as much as the fundamental spectrums.

**Figure 32:**
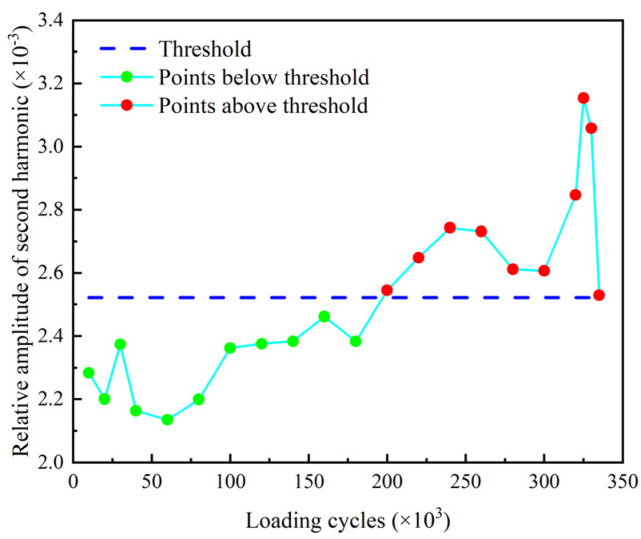
Relative amplitude of second harmonic $\beta_{re}^{\prime }$ versus loading cycles corresponding to 10-cycle excitation signal.

**Figure 33:**
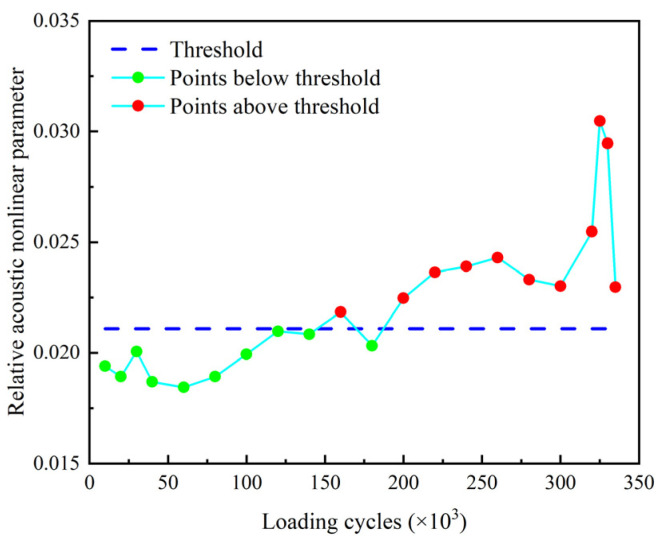
Relative acoustic nonlinear parameter $\beta^{\prime }$ versus loading cycles corresponding to 10-cycle excitation signa.

The nonlinear indexes $\beta_{re}^{\prime }$ and $\beta^{\prime }$ corresponding to the second harmonic corresponding to the 10-cycle excitation signal are shown in Figure 32 and Figure 33. Comparing Figure 32 and Figure 33 with Figure 29 and Figure 30, respectively, it is notable that the variation trends of $\beta_{re}^{\prime }$ and $\beta^{\prime }$ under the two excitation signals with different number of modulation cycles are similar. It can also be found that $\beta_{re}^{\prime }$ and $\beta^{\prime }$ corresponding to the 10-cycle excitation signal are more stable than those corresponding to the 3-cycle excitation signal, especially after their first pass of the thresholds. Meanwhile, both $\beta_{re}^{\prime }$ and $\beta^{\prime }$ corresponding to the 10-cycle excitation signal stably exceed the thresholds after 200 k cycles, which is earlier than those corresponding to the 3-cycle excitation signal. It is also worth noting that both $\beta_{re}^{\prime }$ and $\beta^{\prime }$ corresponding to the 10-cycle excitation signal do not fall below the thresholds after 335 k cycles. This finding indicates that the nonlinear indexes become more sensible to wider cracks, as the number of modulation cycles increase. 

## 5. Conclusions

This paper investigates the feasibility of UGW-based fatigue crack detection of threaded rods through analytical, numerical and experimental studies. The main conclusions that can be drawn on the basis of the studies are given as follows:

(1) The longitudinal L(0, 1) modal guided wave has a simple displacement distribution at low frequency when traveling in the rod. It is easy to be excited and the energy of it is concentrated in the axial direction of the rod. Hence, it is suitable for damage detection of rods.
(2) Via numerical simulations, it is found that the thread-induced cross-sectional changes will cause thread echoes. The linear and nonlinear characteristics of the guided wave obviously change with the growth of cracks, which shows a great potential to use these indexes for damage detection. It is also found that these indexes generally have the highest sensitivity to cracks at central exciting frequency of 60 kHz in the experimental frequency region.
(3) The experiment on UGW-based fatigue crack detection of a threaded rod under cyclic tensile load shows that the reflection coefficient $R_{f}$ is able to warn the crack when it is visible and the accumulative residual squares ARS is able to warn the crack before it is visible. The spectrum-based nonlinear damage indexes, i.e., the relative amplitude of second harmonic $\beta_{re}^{\prime }$ and the relative acoustic nonlinear parameter $\beta^{\prime }$, are generally able to give an earlier warning on the fatigue crack than the linear indexes $R_{f}$ and ARS.
(4) Increasing the number of modulation cycles of excitation signals from 3 to 10 improves the stability of the nonlinear indexes $\beta_{re}^{\prime }$ and $\beta^{\prime }$, and their sensibility to wider cracks.

It should be pointed out that it is mainly a proof-of-concept study based on an ideal case in laboratory. If an in-service threaded rod is considered, there may be impossible to obtain baseline signal from undamaged state and there may be more noise in the echo signals due to the imperfections of the rod and the environment. Improving the proposed approach to be applicable to in-service threaded rods is the ultimate goal of the authors and will be the topic of the authors’ future work. 

## Figures and Tables

**Table 1:** Micro-crack settings of different areas.

Working Condition	C1	C2	C3	C4	C5	CB6
“CAN” area (mm^2^)	11.1	26.1	36.5	49.2	66.9	11.1
Separated area (mm^2^)	0.0	0.0	0.0	0.0	0.0	55.9
